# Long-Term Provision of Acidified Drinking Water Fails to Influence Autoimmune Diabetes and Encephalomyelitis

**DOI:** 10.1155/2018/3424691

**Published:** 2018-06-21

**Authors:** Sundararajan Jayaraman, Arathi Jayaraman

**Affiliations:** ^1^Department of Microbiology and Immunology, University of Illinois at Chicago, 909 South Wolcott Avenue, Chicago, IL 60612, USA; ^2^Department of Surgery, University of Illinois College of Medicine at Peoria, 624 NE Glen Oak Ave, Suite 2675, Peoria, IL 61603, USA

## Abstract

Induction of autoimmune diseases is predisposed by background genetics and influenced by environmental factors including diet and infections. Since consumption of acidified drinking water leads to eradication of gastrointestinal pathogens in animals, we tested whether it may also influence the development of autoimmune diseases. The frequency of spontaneously occurring type 1 diabetes in female NOD mice that were maintained on acidified drinking water by the vendor did not alter after switching to neutral water in our facility. In addition, experimentally induced autoimmune encephalomyelitis was also unaffected by the pH of the drinking water. Interestingly, administration of complete Freund's adjuvant alone or emulsified with a neuronal peptide to induce neurodegenerative disease during the prediabetic stage completely prevented the onset of diabetes regardless of the pH of the drinking water. However, exposure to microbial products later in life had only a partial blocking effect on diabetes induction, which was also not influenced by the ionic content of the drinking water. Taken together, these data indicate that the onset of autoimmune diseases is not influenced by the gastrointestinal pathogen-depleting treatment, acidified drinking water. Thus, administration of acidic drinking water does not appear to be an option for treating autoimmune diseases.

## 1. Introduction

Among the 80 types of autoimmune diseases known (https://medlineplus.gov/autoimmunediseases.html), type 1 diabetes (T1D) and multiple sclerosis (MS) represent as common organ-specific autoimmune diseases. Whereas T1D is diagnosed very early in life, sometimes within months of birth [1], the median age of MS onset is 29 years and notably 3–5% of patients with MS develop signs of the disease during childhood [2]. Genome-wide association studies have implicated class II human leukocyte antigens (HLA) DR/DQ as well as class I HLA-B in T1D [3]. In addition to the HLA class II DR/DQ, MS is also influenced by class I HLA-A, HLA-B, and HLA-C determinants [4]. The disease targets are distinct in these autoimmune diseases, insulin-producing *β*-cells in T1D, and neurons in MS. Despite these differences, both of these diseases appear to cluster together in certain European populations [5, 6]. North–South gradient for the prevalence of these diseases in northern Europe (Scandinavian countries) has been suggested [7]. However, high incidence of T1D in the Mediterranean island Sardinia appears to be inconsistent with this view [8]. Several environmental factors including infections and diminished vitamin D3 level, which is related to low sunlight availability [9], appear to contribute variously to these diseases. Although the intestinal microbes have been implicated in T1D and MS [10, 11], it is unclear whether they can be manipulated to successfully regulate these autoimmune diseases.

Female nonobese diabetic (NOD) mice are the widely used animal model for studying the mechanisms of T1D and treatment procedures for this disease [12, 13]. Higher cumulative diabetes incidence (90–100%) was reported in female NOD mice maintained on acidic drinking water (pH 2.8–3.1) at the Jackson Laboratory (JAX, https://www.jax.org/), which distributed these mice to a majority of laboratories although Japan also distributed to two centers [12]. At 30 weeks of age, cumulative diabetes incidence was reported to vary widely from 15 to 100% in these various centers and even among litters at one location [12]. Variations in breeders (normoglycemic breeders, diabetic females, and brother-sister pairs), light-dark cycle, nutritional values of feed for breeders and stock, age of breeders, and genetic drift in highly inbred colonies have been attributed to vast differences of diabetes incidence in these colonies. Importantly, JAX controls pathogens (bacteria, viruses, and parasites) in NOD mice by housing them in maximum-barrier facilities under specific pathogen-free conditions and providing with sterilized food and acidic water [12]. Although NOD mice are bred in various local animal facilities for experiments, it is unclear whether the housing conditions are comparable to those of JAX. Varying degrees of “cleanliness” that exist in different animal facilities have been attributed to differing frequencies of diabetes. These variations also contribute to the varying success of the same treatment procedure such as acidified drinking water in different locations [12, 13]. The control of infections in locally bred mice is of paramount importance since some parasites such as pinworm in the founders can be easily passed onto their progeny and are hard to eradicate even under specific pathogen-free conditions (https://www.jax.org/). If these pathogens are kept unchecked, they might serve as triggers of the disease or dampen the progression of T1D.

We have previously reported that female NOD mice procured from JAX and maintained on neutral tap water under specific pathogen-free conditions at the University of Illinois at Chicago (UIC) displayed higher frequency (80–100%) of T1D as those in the vendor's facility [14–16]. This indicates that the overall cleanliness of the animal facility at UIC is comparable to that of JAX and NOD mice do not acquire infections at this facility that might alter their susceptibility to develop T1D. However, when three other laboratories obtained NOD breeders from JAX and bred in their individual facilities, varying effects of transitioning from neutral to acidified drinking water were observed. Whereas the frequency of T1D was lowered in one facility [17], others reported either an increase [18] or no effect [19] in response to treatment with acidic water. It is unclear whether these controversial findings could be attributed to different local environments such as the microbial composition and pathogens.

Prior to the publication of these discordant reports [17–19], we transitioned female NOD mice that were maintained on autoclaved neutral pH tap water to acidified drinking water under specific pathogen-free conditions at the UIC animal facility. This was done as a precaution to minimize the spread of suspected gastrointestinal infections. The underlying rationale was that the long-term acid water consumption eradicated opportunistic infections in broilers and pigs [20, 21] and gut-derived sepsis due to *Pseudomonas aeruginosa* infection in mice [22], and greatly reduced the number of bacterial species isolated from the ileum of immunosuppressed mice compared to normal mice [23] without affecting overall biochemical parameters in rats and rabbits [24]. Interestingly, *in vitro* exposure to low pH also influenced the species composition of the human colonic microbiota [25]. Based on these findings, we postulated that whereas low-pH water consumption may increase the occurrence of autoimmune diseases via eradication of gastrointestinal infections, it may not similarly impact apparently healthy animals. We tested this possibility by comparing the effect of consumption of acidified and neutral water on the induction of T1D and experimental autoimmune encephalomyelitis (EAE), a model for MS in the same genetic environment. Our data indicate that the pH of the drinking water exerted no significant influence on the occurrence of both of these autoimmune diseases in female NOD mice procured from JAX and maintained at the UIC animal facility.

## 2. Materials and Methods

### 2.1. Animals and Maintenance

The Office of Animal Care and Institutional Biosafety of UIC approved the animal protocol. Experiments were conducted in accordance with the National Institutes of Health (NIH) guide for the care and use of laboratory animals. Six- to eight-week-old female NOD/ShiLtj mice purchased from JAX (Bar Harbor, ME) were maintained under specific pathogen-free conditions with standard animal chow and water ad libitum. They were either provided with autoclaved neutral or acidified drinking water prepared by the addition of concentrated HCl to reach a pH of 2.8–3.2.

### 2.2. Diabetes Assessment

Nonfasting glucose level was determined weekly in the morning on tail vein blood using a hand-held Accu-Chek glucometer. Glucose level exceeding 250 mg/dl on two consecutive determinations was considered as diabetic [14–16].

### 2.3. Induction of EAE

Mice were injected s.c. with 0.1 ml of complete Freund's adjuvant (CFA) containing *Mycobacterium tuberculosis* (H37Ra) (Fisher Scientific) and emulsified with an equal amount of phosphate buffered saline in some experiments. To induce EAE, mice were immunized s.c. with 100 *μ*g of mouse myelin oligodendrocyte glycoprotein 35-55 (MOG_35-55_) peptide (MEVGWYRSPFSRVVHLYRNGK, Tocris Bioscience) emulsified in 0.1 ml of CFA with an additional 4 mg/ml of *Mycobacterium tuberculosis* (H37Ra) as described [26, 27]. Mice also received 300 ng of pertussis toxin (List Biological Laboratories) i.v. on the day of immunization and 2 days later. EAE score was assigned as follows: 0, no symptom; 1, limp tail; 2, one hind limb weakness; 3, both hind limb weakness; 4, one or both fore limb weakness; and 5, paralysis, moribund, or death.

### 2.4. Statistics

Statistical significance between experimental groups was assessed using the unpaired two-tailed Student's *t*-test (GraphPad Prism 6.0).

## 3. Results and Discussion

### 3.1. Failure of Acidified Drinking Water to Influence the Development of T1D

Female NOD mice maintained on acidic water from birth at JAX were purchased at the age of 6–8 weeks. After their arrival at the animal facility of UIC, they were transitioned to autoclaved neutral pH water, fed with standard animal chow ad libitum, and maintained under specific pathogen-free conditions. The incidence of T1D under these conditions was published previously [14–16]. The incidence of spontaneously occurring T1D was as high as 80–100% in 6 out of 7 experiments (*n* = 30). The only exception was a batch of mice (*n* = 5) in which 60% developed diabetes as late as 30 weeks of age. Thus, female NOD mice maintained on acidic water in JAX and transitioned to neutral water at UIC were similarly susceptible to the development of spontaneous diabetes as in JAX. This indicated the lack of influence of the pH of the drinking water on diabetogenesis.

However, reports indicated that acidic water consumption decreased [17] or increased [18] the diabetes incidence in locally bred mice. Another center reported no effect on diabetes incidence by exposure to acidic water [19]. Paradoxically, all of these three centers obtained breeding pairs from JAX and maintained the offspring on neutral tap water. Many reasons could account for the contrasting effects of acidic water consumption on spontaneously occurring diabetes. Eradication of gastrointestinal infections by exposure to acidic drinking water as reported earlier [20–24] may enhance T1D incidence. A similar explanation is not applicable to reduced diabetes incidence in a different mouse colony [17]. It is possible that depletion of acid-sensitive commensals that might contribute to protection against diabetes may explain this phenomenon. However, neither decrease nor increase [17, 18] in diabetes frequency was reproduced in other studies including ours [14–16, 19].

**Figure 1:**
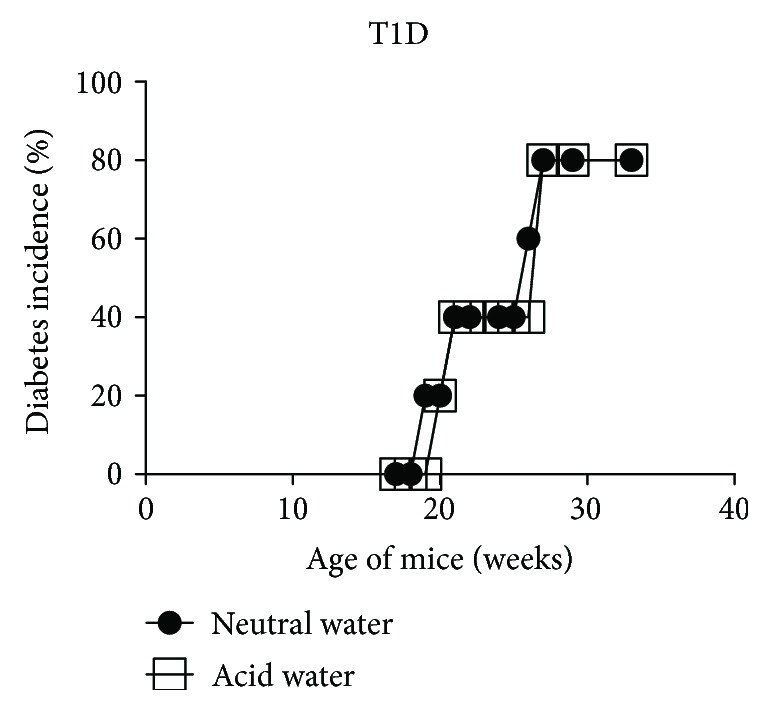
The lack of effect of the pH of the drinking water on T1D development. Littermates of female NOD mice were maintained on neutral or acid water and diabetes was monitored weekly. *n* = 5 per group.

**Figure 2:**
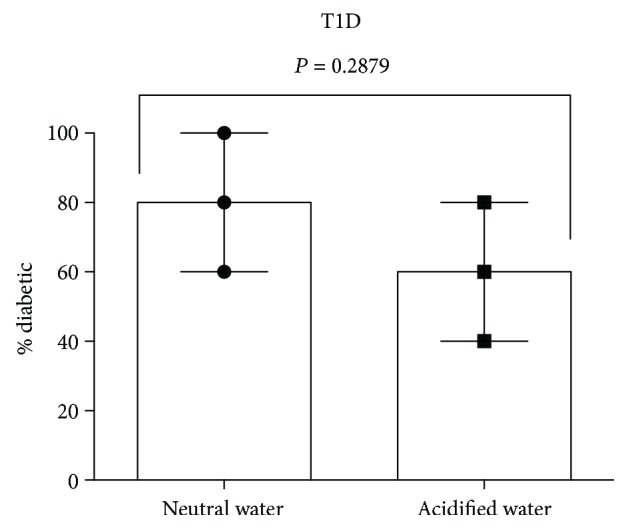
The pH of the drinking water did not influence T1D. Littermates of female NOD mice were maintained either on neutral or acidified water and diabetes was monitored weekly until 30 weeks of age. Individual data points from three independent experiments are indicated. Each data point represents five mice. Mean ± SE for three experiments are shown. *P* value was calculated using the unpaired two tailed Student's *t*-test.

Since the lack of uniform effect of the pH of the drinking water on diabetes incidence was observed in mice that were commercially procured [14–16] or bred in local animal facilities [17–19], it appears that differing conditions of breeding and maintenance at these various locations may substantially contribute to the disease outcome. In order to minimize differences that influence the pathogenesis of these diseases, we made a direct comparison of acidic and neutral water consumption in NOD mice procured from JAX. Upon arrival, the littermates were randomly divided into two groups each consisting of five mice. One group continued to receive the acidified water as in the vendor's facility. The other group was transferred to neutral water and T1D was monitored. Data from a representative experiment shown in Figure 1 indicated that the pH of the drinking water did not influence the frequency or tempo of T1D development. To assess the reproducibility of these results, we performed three more independent experiments at different time points (15 mice per experimental group). The incidence of diabetes in mice maintained on acidic water ranged from 40 to 80%, whereas those transferred to neutral water exhibited slightly increased frequency, 60% to 100% (Figure 2). This is consistent with the lack of significant influence of acidic water treatment on diabetes induction in 8-week-old female NOD mice purchased from JAX [18] and those bred at the NIH [19]. Taken together, these data indicate that consumption of acidic water has no significant impact on diabetes development in NOD mice that were bred under various conditions at JAX and NIH and maintained at distinct locations such as UIC, JAX, and NIH.

### 3.2. Lack of Influence of Acidified Drinking Water on EAE Induction

**Figure 3:**
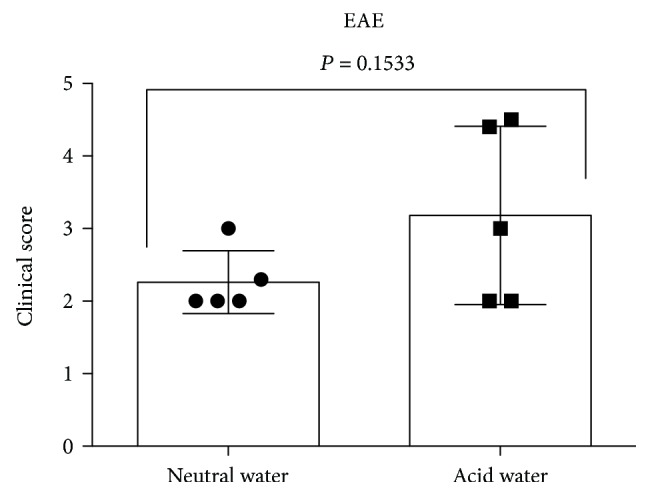
Lack of influence of the pH of the drinking water on EAE. Adult mice provided with neutral or acid water were immunized with MOG_35-55_ to induce EAE. Clinical scores shown are mean ± SE for the 30-day time point (*n* = 5 mice/group). The statistical significance was calculated using the unpaired two-tailed Student's *t*-test.

We next examined whether the change in the pH of the drinking water could influence the induction of another autoimmune disease, EAE in the same genetic environment. To this end, 6–8-week-old female NOD mice were procured from JAX and continued on acidified drinking water. Littermates were also transitioned to neutral water. After two weeks of acclimatization, both groups of mice were immunized with MOG_35-55_ emulsified in CFA to induce EAE as described [26, 27]. Data shown in Figure 3 indicate that the severity of the clinical disease after 30 days of immunization was slightly but not significantly decreased when mice were transitioned from acidic to neutral water. Acid water consumption also did not influence the frequency of the disease occurrence since 100% of immunized NOD mice in both groups developed EAE (not shown). These results validate the lack of influence of acid water consumption on the development of experimentally induced MS-like disease (EAE) as naturally occurring T1D in the same genetic background.

### 3.3. Subversion of T1D by Antimicrobial Immune Responses Is Time Dependent and Unaffected by the pH of the Drinking Water

**Figure 4:**
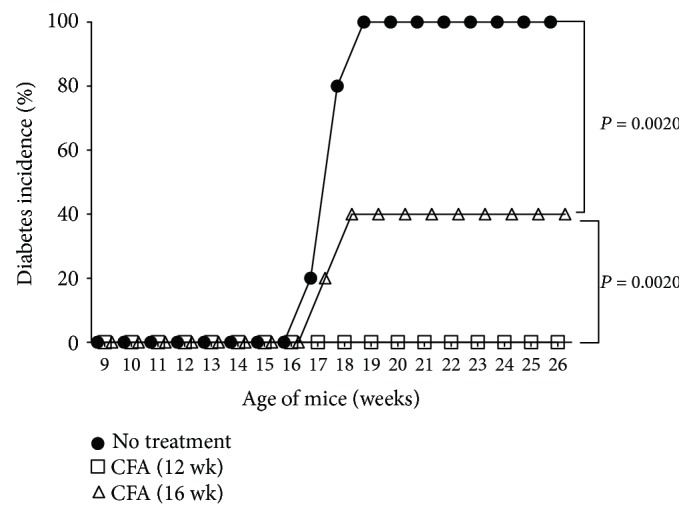
Differential influence of CFA on diabetes development. Prediabetic (12-week-old) female NOD mice or older mice (16 weeks) were either treated with CFA or left untreated and maintained on neutral tap water. Cumulative diabetes incidence is shown for untreated (*n* = 10) and those treated with CFA at 12 (*n* = 5) or 16 weeks (*n* = 5) of age. Statistical significance was calculated using the unpaired two-tailed Student's *t*-test.

It has been shown that exposure to microbial products such as *Mycobacterium tuberculosis H37Ra* in complete Freund's adjuvant (CFA) during prediabetic stage prevented the onset of T1D in rats [28] and NOD mice [29–34]. Consistently, we also observed 100% protection against T1D when prediabetic (12-week-old) mice were injected with CFA and maintained on neutral tap water (Figure 4). Several mechanisms including induction of Mac-1^+^ suppressive macrophages [29], T regulatory cells [30, 33], NK [32], and NKT cells [34] have been implied in CFA-mediated protection against T1D in NOD mice. Interestingly, CFA or BCG administration significantly reduced the incidence of diabetes in NOD mice characterized by nondestructive inflammation of the pancreas [31]. However, when CFA administration was delayed until 16 weeks of age, protection against T1D was considerably reduced from 100 to 60% (Figure 4). It appears that the T regulatory cells, NK and NKT cells, and suppressive macrophages elicited in adult life by mycobacterial antigens [29–34] could not effectively reverse extensive *β*-cell destruction in overtly diabetic mice. These results suggest that antimycobacterial immune responses tend to lose efficacy in subverting autoimmune diabetes beyond a critical stage in life of the NOD mouse.

It was of interest to examine whether the lack of complete protection against T1D induced by immune responses elicited against mycobacterial antigens late in life could be modulated by the pH of the drinking water. Data shown in Table 1 indicate that immunization of prediabetic (12-week-old) NOD mice maintained on neutral pH water dramatically reduced the T1D incidence to 6.6% for as long as 28 weeks of age, consistent with the data presented in Figure 4. Provision of acidic water did not reduce the level of protection against T1D induced by CFA administration (5.0%, Table 1). However, as observed earlier (Figure 4), delaying the administration of CFA till 16 weeks of age remarkably diminished the degree of protection against diabetes by 55% in mice administered with neutral water (Table 1). Switching to acidified drinking water did not influence the partial protection afforded by CFA administration (Table 1). Interestingly, EAE induction remained unaltered regardless of the time of immunization (prediabetic versus late-stage T1D) or the source of drinking water (Table 2). Taken together, these data indicate that the blockade of T1D is dependent on the timing of elicitation of immune responses to mycobacterial products and thereby on the underlying immune status rather than the ionic content of the drinking water. Inasmuch as acidic drinking water can modulate the gut luminal microbiota [20–25], the lack of influence of low-pH drinking water on T1D and EAE rules out a role for acid-sensitive gut-resident microbes in the pathogenesis of these autoimmune diseases under specific pathogen-free conditions.

## 4. Conclusions

The data presented herein demonstrate that long-term provision of acidified drinking water had no substantial influence on the onset of autoimmune diseases such as diabetes and MS-like disease in female NOD mice. Importantly, acid water treatment also failed to compensate compromised protection against T1D by the elicitation of mycobacterial immune responses in older mice. Although long-term provision of acidic drinking water is known to eradicate gastrointestinal infections and reduce the colonic bacterial load [20–25], it does not appear to be linked to the onset of either T1D or EAE. Therefore, acidified water feeding is not a viable option for treating autoimmune diseases. Rather, it could have adverse effect (exacerbation) in those who have underlying gastrointestinal infections. Thus, caution should be exercised before implementing acidic water treatment for clinical applications.

## Figures and Tables

**Table 1:** Lack of influence of acidified drinking water on diabetes induction in older mice regardless of exposure to mycobacterial antigens.

Treatment	12 weeks CFA	16 weeks CFA
Neutral water	6.6 ± 6.6% (0%, 0%, 20%) (*n* = 15)	45 ± 5.0% (40%, 50%) (*n* = 10)^∗^
Acidic water	5.0 ± 5.0% (0%, 10%) (*n* = 10)	45 ± 8.3% (40%, 50%, 60%) (*n* = 15)^∗∗^

Mean ± SE of diabetes incidence is shown. Diabetes incidence in individual experiments and numbers of mice are shown in parentheses. *P* values as determined using the unpaired two-tailed Student's *t*-test between 12- and 16-week time points in corresponding groups were ^∗^*P* = 0.0265 and ^∗∗^*P* = 0.0124.

**Table 2:** Lack of influence of acidified drinking water on EAE induction during various stages of life.

Treatment	MOG_35-55_ + CFA-12 weeks	MOG_35-55_ + CFA-18 weeks
Neutral water	2.3 ± 0.3 (3.0, 2.0, 2.0) (*n* = 15)	2.5 ± 0.5 (2.0, 3.0) (*n* = 10)^+^
Acidic water	3.2 ± 1.25 (4.5, 2.0) (*n* = 10)	3.13 ± 0.69 (4.4, 3.0, 2.0) (*n* = 15)^++^

Mean ± SE of clinical scores after 30 days of immunization is shown. Individual values and numbers of mice are shown in parentheses. *P* values as determined using the unpaired two tailed Student's *t*-test between 12- and 16-week time points in corresponding groups were ^+^*P* = 0.788 and ^++^*P* = 0.93.
